# Anti-osteoarthritis potential of peppermint and rosemary essential oils in a nanoemulsion form: behavioral, biochemical, and histopathological evidence

**DOI:** 10.1186/s12906-021-03236-y

**Published:** 2021-02-09

**Authors:** Mojgan Mohammadifar, Mohammad Hossein Aarabi, Fatemeh Aghighi, Maryam Kazemi, Zarichehr Vakili, Mohammad Reza Memarzadeh, Sayyed Alireza Talaei

**Affiliations:** 1 Biochemistry and Nutrition in Metabolic Diseases Research Center, Institute for Basic Sciences, Kashan University of Medical Sciences, Kashan, Iran; 2 Department of Biochemistry, Faculty of Pharmacy, Isfahan University of Medical Sciences, Isfahan, Iran; 3 Physiology Research Center, Institute for Basic Sciences, Kashan University of Medical Sciences, Kashan, Iran; 4 Department of Pathology, School of Medicine, Kashan University of Medical Sciences, Kashan, Iran; 5 Barij Essence Medicinal Plants Research Center, Kashan, Iran

**Keywords:** Nanoemulsion, Essential oil, *Mentha piperita*, *Rosmarinus officinalis* L., Osteoarthritis, Rat

## Abstract

**Background:**

This study aimed to evaluate the effect of nanoemulsion containing peppermint and rosemary essential oils in rats with osteoarthritis (OA).

**Methods:**

In this experimental study, we prepared a nanoemulsion containing peppermint and rosemary essential oils by spontaneous emulsification and evaluated the nanoemulsion’s dermal irritation and toxicity. Investigating the analgesic effect of the nanoemulsion, we randomly assigned 36 male rats to 6 groups: Control (saline injection into the knee), osteoarthritis (intra-articular injection of 2 mg monosodium iodoacetate), and four groups of OA treated with nanoemulsion gel, nanoemulsion solution, rosemary and peppermint essential oil gel, or diclofenac sodium. Treatments were administered topically at a dose of 1 ml daily. Using behavioral tests, we assessed pain on days 1, 4, 7, and 14 after injection. Finally, we did the histopathological and biochemical evaluation of rats’ knee joints.

**Results:**

There were no irritation signs on the animals’ skin after receiving the nanoemulsion and no changes in the hematological and biochemical parameters of rats’ blood compared to the control group. Receiving nanoemulsion decreased the mechanical (*P* < 0.001) and thermal allodynia (*P* < 0.05), thermal hyperalgesia (*P* < 0.05), and ambulatory-evoked pain in comparison with the OA group. Also, the nanoemulsion receiving rats showed an increase in SOD and GPx activity and a decrease in MDA level. Histopathology of synovial tissues confirmed the results of behavioral and biochemical tests.

**Conclusion:**

The nanoemulsion containing essential oils of peppermint and rosemary reduces osteoarthritis pain via increasing antioxidant capacity and improving the histopathological features of the rats’ knee joint.

## Background

Osteoarthritis (OA) is the most common musculoskeletal disorder characterized by the progressive destruction of articular cartilage, local inflammation, and joint pain [1]. The main complaint of patients is debilitating pain, which influences their quality of life [2], and it gradually becomes more resistant to treatment [3]. Since no disease-modifying drugs are available, treatments’ main goals are symptomatic such as analgesic and anti-inflammatory drugs [2]. Although these treatments effectively reduce pain, inflammation, and symptoms of the OA, but do not have a significant impact on the prevention of the disease progression [4]. Knowledge in herbal medicine and its use has increased in recent years due to higher safety and a reasonable price. The effect of the various herbs on osteoarthritis treatment and prevention has been investigated.

*Mentha piperita* (peppermint), a natural hybrid between *Mentha spicata* and *Mentha aquatica*, [5] contains about 1.2–1.5% of essential oil, and menthol, as its main active ingredient, has various therapeutic effects such as anti-inflammatory, anti-oxidant, anti-bacterial, anti-cancer, and analgesic effects [6]. Taher has revealed that peppermint can reduce the writhing number induced by acetic acid injection in mice [7]. Using acetic acid-induced nociception and carrageenan-induced paw edema tests, Kehili et al. have proved the anti-nociceptive and anti-inflammatory effects of peppermint essential oil in rats [8]. Also, Belemkar and colleagues have shown analgesic effects of *Mentha piperita* methanolic extract by both the hot-plate and tail immersion tests in rats [9]. A study showed that topical application of a cream containing *Mentha piperita*, *Cinnamomum camphora*, and *Pinus roxburghii* essential oil reduces pain and swelling in patients with osteoarthritis [10].

In traditional medicine, *Rosmarinus officinalis* L. (rosemary) uses as a treatment for asthma, renal colic pain, and dysmenorrhea. The main components isolated from rosemary essential oil monoterpenes have anti-inflammatory, anti-oxidant, and anti-nociceptive effects [11]. Raskovic et al. have demonstrated that rosemary essential oil can alleviate pain in the hot-plate test [12]. Also, Belkhodja et al. have studied the effect of rosemary and white poplar essential oils in an animal model of knee osteoarthritis. Radiographic and histologic data of this study have shown that the essential oil has a protective effect against osteoarthritis in rats [13]. Ghasemzadeh and colleagues have evaluated the anti-allodynic and anti-hyperalgesic effects of rosemary extract in rats with neuropathic pain. This study also has demonstrated that rosemary extract has anti-inflammatory and anti-apoptotic effects that can treat neuropathic injury [14]. Besides, an in vitro study on bovine articular cartilage cells has revealed that rosemary extract slows down cartilage degeneration [15].

Despite the medicinal properties of essential oils, some factors such as volatility, sensitivity to light and oxygen, and hydrophobicity have restricted the use of essential oils in pharmaceutical formulations [16]. Nanoemulsion-based delivery systems are favorite methods for improving the stability, decreasing volatility, and enhancing water solubility of the essential oils [17]. The nanoemulsification of essential oils can increase their stability and improve antimicrobial efficacy [17]. Nanoemulsions are transparent and thermodynamically stable dispersion of oil and water balanced by an interfacial layer of surfactant and co-surfactant molecules. The droplet size in nanoemulsion is approximately 10–200 nm. The tiny size of nanoemulsion droplet provided greater surface area, enhancing skin absorption and making these systems suitable for transdermal delivery. The nanoemulsion improved the bioavailability of the drug and increased the solubility of the lipophilic agent [18].

Due to the medicinal properties of peppermint and rosemary essential oils and their limitations as a drug, the present study aimed to evaluate the effect of nanoemulsion containing rosemary and peppermint essential oils in a rat model of osteoarthritis.

## Methods

### Nanoemulsion preparation

Rosemary and peppermint essential oils were purchased from the Barij-Essence pharmaceutical company (Kashan, Iran). A spontaneous emulsification technique was used to make the nanoemulsion. To achieve the best formulation for a stable nanoemulsion, various ratios of emulsifiers and essential oils were examined (results not published). The results showed that the best ratio for emulsifiers is 1: 1: 3 for crudert, tween 20 and tween 80, respectively. Also, the optimum ratio of propylene glycol and polyethylene glycol 400 as co-solvent was obtained 1:2. To prepare nanoemulsion, the aqueous phase was assisted by solvents and water, and an oily phase consisting of emulsifiers and essential oils were prepared separately. The beaker was sealed with parafilm and placed into bain-marie at 35 °C. In the next step, the aqueous phase was added dropwise to the oil phase, which was placed in a hot water bath on a heater stirrer (37 °C, 200 RPM). The homogenization process continued for 20 min at high speed to achieve a single-phase, transparent solution. The resulting solution was transferred to a dark container and stored at an appropriate temperature.

### Animals

Healthy and adult male and female Wistar rats (12 weeks old, 200–220 g) and New Zealand white male rabbits (15 weeks old, 2200–2500 g) were purchased from Tehran Pasteur Institute. Animals were individually housed in standard cages in the controlled environmental situation (temperature 22 ± 2 °C, humidity 40–50%). They were kept in 12 h dark/light cycle and had free access to water and food. All experimental procedures were carried out in accordance with the *Directive 2010/63/EU* on the protection of animals used for scientific purposes and approved by the ethical committee of Kashan University of Medical Science, Kashan, I.R. Iran (IR.KAUMS.MEDNT.REC.1396.76).

### Acute irritation study

Acute irritation study in rabbit was conducted following Organization for Economic Co-operation and Development (OECD) guideline number 404 with a sequential testing strategy on three healthy white rabbits (1.2–1.5 kg) [19]. Approximately 24 h before the test, the dorsal fur of animals was removed with an electrical clipper. Care was taken to avoid irritating the skin, and only rabbits with intact skin were used.

Initial test: One patch of the nanoemulsion was placed on the dorsal area of a rabbit’s trunk and removed after 3 min. If no serious skin reaction was observed, the second patch was applied to a different site and removed after 1 h. If the observation at this stage indicated that exposure could humanly be allowed, the third patch was applied and then removed after 4 h. The rabbit was examined for 14 days for any skin changes.

Confirmatory test: If serious skin reaction was not observed in the initial test, irritant or negative response was confirmed using up to two additional animals. One patch of the nanoemulsion was applied on the dorsal side of each animal’s trunk for an exposure period of 4 h. The animals were observed for 1, 24, 48, 72 h(s) and later for 14 days after removal of patches and examined for erythema, edema, and scar.

### Acute dermal toxicity study

Acute dermal toxicity test was performed following the guideline of OECD number 402 [20]. This study was done on two groups of animals: control and nanoemulsion receiving groups. Each group included three male and three female rats were kept individually. Approximately 24 h before the test, fur was removed from the trunk’s dorsal area with an electrical clipper. Care was taken to avoid irritating the skin, and only rats with intact skin were used. A dose of 2000 mg of the nanoemulsion was applied to 10% of animals’ body surface area and covered with gauze patch and fixed with cloth glue for 24 h. The residual test substance was removed after the exposure period. Then, animals’ skin was studied at 30 min and 1, 2, and 4 h(s) for edema, erythema, and any other types of dermal change. Animals were kept under observation for any changes in the skin, eyes, mucus membranes, behavior patterns, diarrhea, salivation, tremor, and mortality for 14 days post-exposure. Body weight was recorded on days 0 (before treatment), 7, and 14. At the end of the study, animals were anesthetized and sacrificed for blood sampling. Biochemical and hematological parameters were detected.

### Induction of osteoarthritis

Evaluating the anti-nociceptive effects of the nanoemulsion, osteoarthritis was induced by intra-articular injection of monosodium iodoacetate (MIA). Briefly, rats were anesthetized with Ketamine-Xylazine (100–10 mg/kg, I.P.) (Alfasan, The Netherlands) and 2 mg of MIA (Sigma-Aldrich, USA) dissolved in 25 μl sterile normal saline was injected into the left knee joint with a 27-gauge needle inserted through the intra-patellar tendon. Control animals were given a single injection of the vehicle into the left knee joint [21]. In this study, 36 rats were randomly divided into six groups (*n* = 6 for each) including Control (CO), Osteoarthritis (OA), Osteoarthritis received gel contained the nanoemulsion (NG), Osteoarthritis received gel contained Mentha and Rosemary essential oils (EG), Osteoarthritis received the nanoemulsion solution (NS), and Osteoarthritis received gel contained 1% diclofenac sodium (Razak Co., Iran) (DG). Gels and solutions were topically applied at a dose of 1 ml on injected knee daily for 14 days.

### Behavioral studies

Behavioral tests were done at the days 1, 4, 7, and 14 after induction of osteoarthritis as follows:

#### Mechanical allodynia (von-Frey test)

Mechanical allodynia was evaluated by measuring the hind paw withdrawal response to von-Frey filament stimulation (with increasing forces ranging from 2 to 60 g, Stoelting Inc., Wood Dale, IL). Rats were placed in a plexiglass cage with a mesh floor and allowed to acclimate for 15 min or until exploratory behavior ceased. Filaments were applied at a 90° to the mild plantar of the rat’s left hind paw (ipsilateral side of MIA injection). The stimulation was used three times consecutively by pushing down on the hind paw until the rat withdrew its paw or the fiber bowed. Lifting the paw due to normal locomotor behavior was ignored. The smallest filament size, which evoked at least two withdrawal responses during three consecutive applications, was considered as the withdrawal threshold. Each filament was applied approximately 1 s, and intervals between each stimulation were about 1 min [22].

#### Cold allodynia (acetone test)

Cold sensitivity was quantified by acetone spray test (evaporation-evoked cooling). Rats were placed on a wire mesh floor, and one drop of acetone was applied to the plantar surface of the hind paw using a propylene tube attached to the syringe (5 times at 5 min intervals). Paw withdrawal frequency was reported as percentage (the number of paw withdrawal/number of trials× 100) [22].

#### Thermal hyperalgesia (hot-plate test)

Paw withdrawal latency in response to radiant heat was measured using the plantar test apparatus (UgoBasile, Varese, Italy). Rats were placed within plexiglass on a transparent glass floor. An infrared ray that constitutes the heat source was moved under the mid-plantar surface of the hind paw (temperature: 52 °C). The latency (seconds) between the heat stimulus onset and paw withdrawal was defined as thermal withdrawal latency. A cut-off time of 22 s was considered to avoid tissue damage. Each paw was tested three times with inter-stimulus intervals. Mean withdrawal latency time for ipsilateral (injected), and contralateral paw was calculated separately [22].

#### Ambulatory-evoked pain

To calculate this index, animals were placed into a plexiglass chamber with a flat floor, and after adaptation to the new environment, the ambulatory-evoked pain was scored from 0 to 3 as follows: 0; no limp, 1; slight limp but no decrease in usage of the ipsilateral limb, 2; limp with a reduction in usage of the ipsilateral limb, and 3; avoidance of usage the ipsilateral limb [21].

#### Tissue collection

On day 15, rats were euthanized by intraperitoneal injection of 200 mg/kg sodium thiopental (Exir Pharmaceutical Company, Iran), and the left knee joint immediately was dissected. Half of the samples (3 animals in each group) were fixed in 10% neutral buffered formalin for pathological investigation. The other pieces were snap-frozen in liquid nitrogen and then stored at − 70 °C for biochemical studies.

### Biochemical studies

The oxidative stress parameters were evaluated in tissue homogenates. Briefly, 0.2–0.3 g of each tissue sample was homogenate with ice-cooled KCl (150 mM), and then the mixture was centrifuged (3000 g for 10 min). Determining lipid peroxidation, the MDA content of the sample was measured using thiobarbituric acid (TBA) reaction as follows: 0.5 ml of homogenate supernatant was mixed with 1 ml trichloroacetic acid (20%) and centrifuged at 2500 g for 10 min, and then 1 ml of TBA 0.067% was added to the sample (0.5 ml of centrifuged solution). The reaction solution was heated 80 for 15 min and then chilled in Ice. The absorbance of the solution was measured spectrophotometrically at 532 nm. The amount of MDA was expressed as nmol/mg protein [23]. Also, the protein content of samples was detected using Lowry et al. method [24]. Superoxide dismutase (SOD) and glutathione peroxidase (GPx) activity were measured by standard laboratory ELISA kit (ZellBio, Germany).

### Histopathological study

After fixation in formalin, joint tissues were transferred to Ethylene-diaminetetrachloroacetic acid (EDTA) in phosphate-buffered (pH: 7.4, 4 °C) until completely decalcified. Samples were processed in a tissue processor and paraffin-embedded. Five micrometers-thick tissue sections were stained with Hematoxylin and Eosin [13].

### Statistical analysis

All results were reported as mean ± SEM. The Mann-Whitney U test was used to analyze the acute dermal toxicity data. Statistical analysis of behavioral and biochemical tests in the OA model was performed by Two-way repeated measures ANOVA test followed by Tukey’s test. All statistical analysis was done using GraphPad Prism software (version 7.05), and *p* < 0.05 were considered as a significant difference.

## Results

### Nanoemulsion did not caused any irritation

In the initial test, we did not see any serious skin reaction after any of the three sequential (3 min, 1 and 4 h(s)) exposures, and also there were no dermal changes after 14 days. So, the confirmatory test was done using up two additional rabbits. Skin reactions were observed for 1, 24, 48, 72 h(s) and later for 14 days after removing patches, and there was no sign of erythema, edema, and scar.

### Nanoemulsion did not caused dermal toxicity

Clinical signs were evaluated throughout the observation for 14 days post-exposure. There was no mortality seen during the toxicity study. Also, no abnormality was observed in the skin, eyes, mucous membranes, and behavioral patterns after nanoemulsion application. Further, no sign of tremors, convulsions, salivation, diarrhea, and coma was observed after nanoemulsion treatment. Besides, there was no significant (*P* > 0.05) difference in body weight in the treatment group compared to the control group. In comparison to the control group, none of the hematological (Table 1) and biochemical (Table 2) parameters changed significantly (*P* > 0.05) after nanoemulsion treatment in both male and female rats.

**Table 1:** Effect of nanoemulsion treatment on biochemical parameters of rats’ serum during the acute dermal toxicity study

	Control		Nanoemulsion	
	Male (*n* = 3) Mean ± SEM	Female (*n* = 3) Mean ± SEM	Male (*n* = 3) Mean ± SEM	Female (*n* = 3) Mean ± SEM
WBC	11.13 ± 0.23	9.07 ± 0.35	11.27 ± 0.32	8.83 ± 0.27
RBC	7.72 ± 0.95	7.27 ± 0.15	7.73 ± 0.18	7.43 ± 0.2
HGB	14.37 ± 0.12	13.23 ± 0.15	14.37 ± 0.2	13.37 ± 0.2
HCT	46.2 ± 1.33	42.73 ± 1.27	45.73 ± 1.78	42.9 ± 1.5
MCV	54.37 ± 1.21	52.33 ± 0.85	54.73 ± 1.16	52.17 ± 1.13
MCH	17.03 ± 0.88	17.7 ± 0.35	17.2 ± 0.21	18.1 ± 0.25
MCHC	33 ± 0.23	33.27 ± 0.55	32.77 ± 0.65	34.43 ± 0.87
PLT	1085.33 ± 20.54	1063.67 ± 20.04	1099.33 ± 8.57	1082.33 ± 17.23
LYM	67.33 ± 2.96	64 ± 0.58	66.33 ± 1.2	62.67 ± 1.2
NEUT	28 ± 2.89	31 ± 1.53	29.67 ± 0.67	32.33 ± 1.2
MONO	3 ± 0.58	3 ± 0.58	2.33 ± 0.33	3 ± 0.58
EOS	1.67 ± 0.33	2 ± 0.58	1.67 ± 0.33	2 ± 0.58

There was no significant difference in hematological parameters between nanoemulsion treated group and control group in male and female rats

*WBC (× 1000/mm*^*3*^*)* White blood cells, *RBC (× 1000/mm*^*3*^*)* Red blood cells, *HGB (g/dl)* Hemoglobin, *HCT (%)* Hematocrits, *MCV (fl)* Mean corpuscular volume, *MCH (pg)* Mean corpuscular hemoglobin, *MCHC (g/dl)* Mean corpuscular hemoglobin concentration, *PLT (× 1000/mm*^*3*^*)* Platelets, *LYM (%)* Lymphocytes, *NEUT (%)* Neutrophils, *MONO (%)* Monocytes, *EOS (%)* Eosinophils

**Table 2:** Effect of nanoemulsion treatment on hematological parameters of rats during the acute dermal toxicity study

	Control		Nanoemulsion	
	Male (*n* = 3) Mean ± SEM	Female (*n* = 3) Mean ± SEM	Male (*n* = 3) Mean ± SEM	Female (*n* = 3) Mean ± SEM
TG	82.67 ± 1.45	76.33 ± 0.88	83.33 ± 0.88	75.67 ± 1.76
CHOL	112 ± 1.53	119.67 ± 0.88	115 ± 0.58	120.33 ± 2.6
LDL	39 ± 0.58	43.33 ± 0.88	40.33 ± 0.88	44.33 ± 1.76
HDL	59 ± 0.58	65.33 ± 1.2	61.67 ± 0.33	65 ± 1.73
UA	0.36 ± 0.01	0.53 ± 0.03	0.37 ± 0.01	0.52 ± 0.01
TP	5.4 ± 0.12	5.7 ± 0.15	5.67 ± 0.2	5.57 ± 0.09
CRE	0.8 ± 0.06	0.91 ± 0.05	0.73 ± 0.09	0.87 ± 0.09
ALT	46.67 ± 0.88	41.33 ± 1.45	47 ± 1.15	41 ± 1.15
AST	34.33 ± 0.88	31 ± 0.58	32.33 ± 0.88	35 ± 1.15

There was no significant difference in biochemical parameters between nanoemulsion treated group and control group in male and female rats

*TG (mg/dl)* Triglyceride, *CHOL (mg/dl)* Total cholesterol, *LDL (mg/dl)* Low density lipoprotein, *HDL (mg/dl)* High density lipoprotein, *UA (mg/dl)* Uric acid, *TP (g/dl)* Total protein, *CRE (mg/dl)* Creatinine, *ALT (IU/L)* Alanine aminotransferase, *AST (IU/L)* Aspartate aminotransferase

### Behavioral studies

#### Nanoemulsion decreased mechanical allodynia

**Fig. 1:**
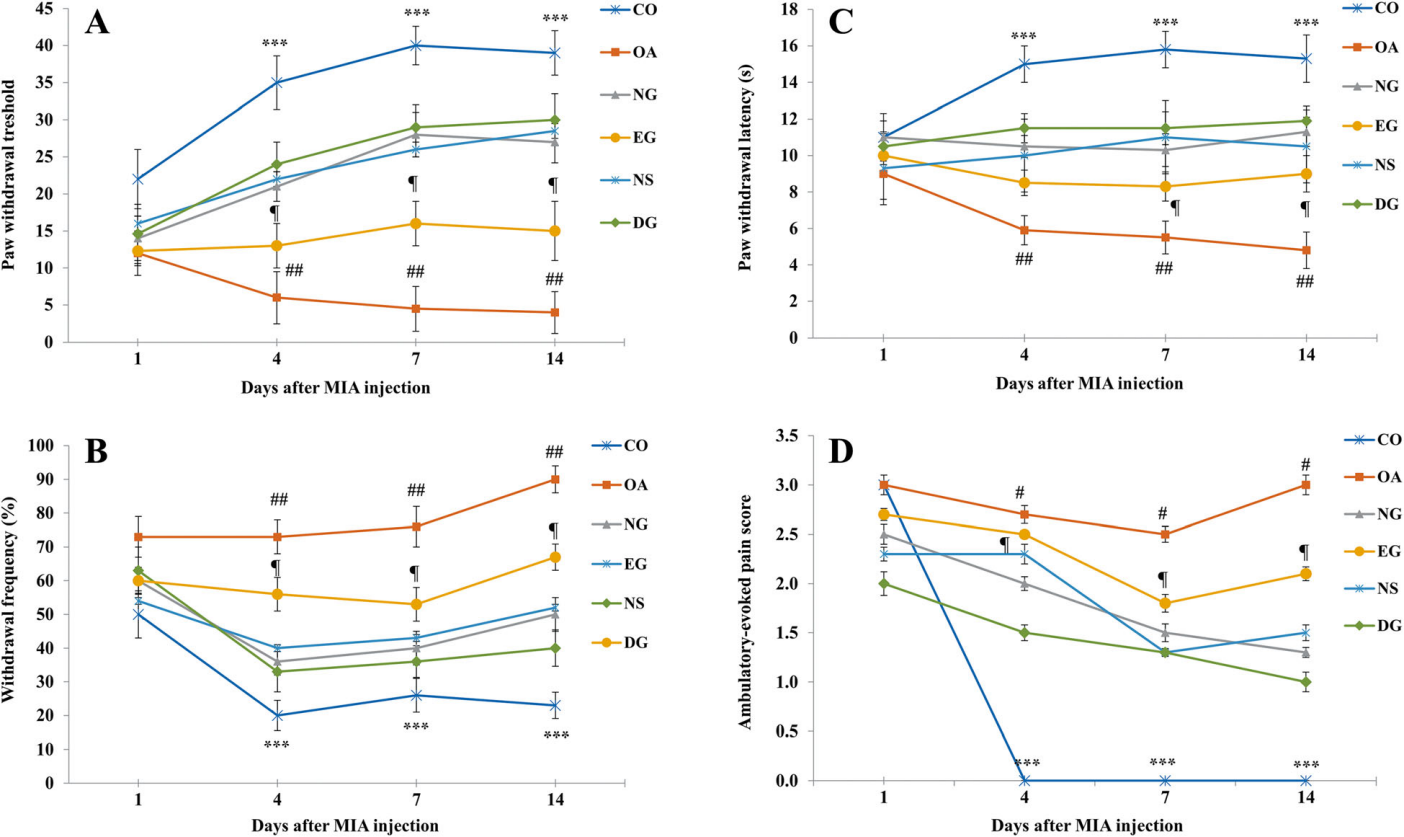
The response to von-Frey filaments stimuli (paw withdrawal threshold; PWT) (**a**), acetone stimuli (paw withdrawals frequencies; PWF) (**b**), and noxious radiant heat (paw withdrawal latencies; PWL) (**c**) was measured at days 1, 4, 7, and 14 after the injection of MIA in all groups of the study (*n* = 6 in each group). PWT, PWF, and PWL were significantly decreased from the first day after MIA injection into the knee joint to the end of study. MIA injection caused a decrease in PWT, PWF, and PWL compared to the CO group. Also, the NG, EG, NS, and DG treatments significantly increased PWT, PWF, and PWL compared to the OA group on days 4, 7, and 14 after the injection. Also, the ambulatory-evoked pain was scored on days 1, 4, 7, and 14 for all animals (**d**). All the animals on the first day fell due to inflammation and pain caused by intra-articular injection. MIA injection caused a significant decrease in the limb use. All the treatments increased the limb use score in comparison to the OA group. The ambulatory-evoked pain was scored from 0 to 3 as follows: 0; no limp, 1; slight limp but no decrease in usage of the ipsilateral limp, 2; limp with decrease in usage of the ipsilateral limp, and 3; avoidance of usage the ipsilateral limp. Data are expressed as mean ± SEM. *** *P* < 0.001 compared to the CO group, ## *P* < 0.01 compared to the OA group, and ¶ *P* < 0.05 the NG vs. the EG group

MIA injection to the knee joint led to a significant decrease in the withdrawal threshold in comparison with the CO group (*P* < 0.001). Paw withdrawal thresholds to von-Frey hairs were significantly decreased from the first day after MIA injection into the left knee joint to the end of the study. Treatment with the gel contained the nanoemulsion (NG), gel contained peppermint and rosemary essential oils (EG), and gel contained 1% diclofenac sodium (DG) increased withdrawal threshold compared to the OA group. The difference between the paw withdrawal thresholds in the OA group and the NG, EG, and DG groups on days 4, 7, and 14 after the injection was significant (*P* < 0.01). Data analysis also showed that the difference between the NG group and the DG group was not significant (*P* > 0.05). At the same time, the antinociceptive effect of the NG was significantly more than the EG group (*P* < 0.05) (Fig. 1a).

#### Nanoemulsion decreased cold allodynia

As shown in Fig. 1b, the injection of MIA into the rats’ knee joint caused an increase in withdrawal frequency in comparison to the CO group. The difference between the osteoarthritis and CO groups in all days of the test was statistically significant (*P* < 0.001). All of the treatments significantly reduced acetone’s sensitivity compared to the OA group (*P* < 0.01). The statistical analysis also indicated that the treatment with the gel containing the nanoemulsion was more effective than gel containing essential oil in reducing cold hypersensitivity (*P* < 0.05). Moreover, no significant difference was observed between the NG and DG groups (*P* = 0.06).

#### Nanoemulsion decreased thermal hyperalgesia

Compared to the control rats, MIA’s intra-articular injection caused a significant reduction of paw withdrawal latencies to heat stimuli (Fig. 1c). Statistical analysis showed that the difference between the CO and OA groups on days 4, 7, and 14 after the injection is significant (*P* < 0.001). All treatments significantly increased the withdrawal latency compared to the osteoarthritis group (*P* < 0.01). Also, the data analysis showed that the anti-nociceptive effect of nanoemulsion was higher than essential oil gel (*P* < 0.05), and the difference between the NG and DG groups was not significant (*P* = 0.09).

#### Nanoemulsion decreased ambulatory-evoked pain score

Ambulatory-evoked pain was scored on days 1, 4, 7, and 14 for all study groups. All animals on the first day fell due to inflammation and pain caused by intra-articular injection. A significant decrease in the ipsilateral limb use was observed in the OA group rats, and there was no ambulatory pain in the control rats during the following days. As shown in Fig. 1d, all treatments increased the limb use score compared to the OA group.

### Biochemical studies

#### Nanoemulsion decreased MDA content of the rats’ synovial tissue

**Fig. 2:**
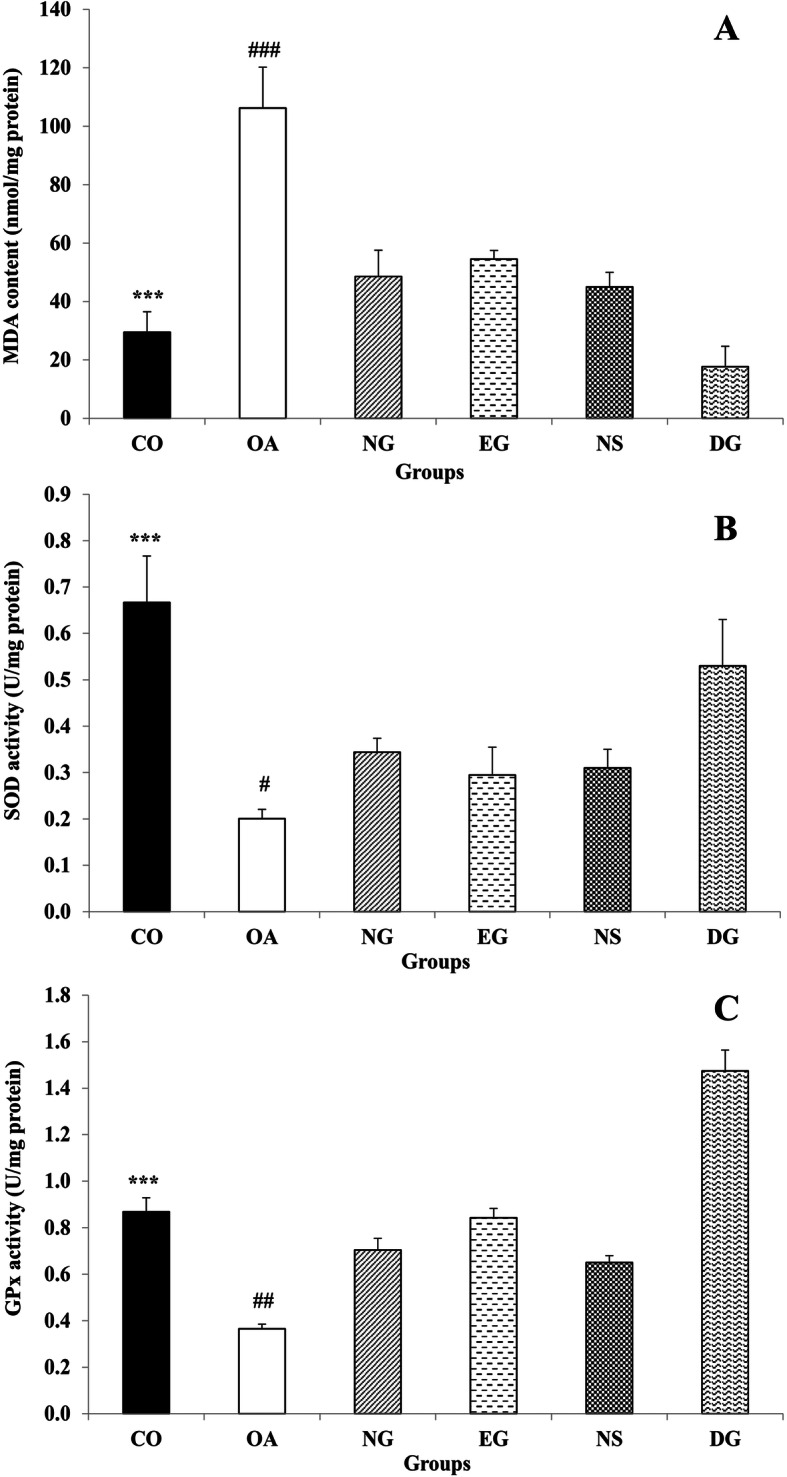
Effect of MIA injection and different treatment on MDA amount (**a**), SOD activity (**b**), and GPx activity (**c**) in the joint tissue of the animals (*n* = 3 in each group). While intra-articular injection of MIA increased MDA content and decreased SOD and GPx activity in the rats’ synovial tissue, all the treatments, especially diclofenac sodium gel, reversed the effects of MIA injection. Data are expressed as mean ± SEM. *** *P* < 0.001 the CO vs. the OA group, # *P* < 0.05 all the treatments groups vs. the OA group, ## *P* < 0.01 all the treatments groups vs. the OA group, ### *P* < 0.001 all the treatments groups vs. the OA group

In order to highlight the impact of MIA injection on lipid peroxidation, the concentration of MDA in synovial tissue was determined (Fig. 2a). The obtained results showed that MDA level of synovial tissue in the OA group significantly elevates in comparison to the CO group. The MDA level of the NG, EG and DG groups was lower than the OA group (*P* < 0.001) but the lowest amount of MDA was found in DG group.

#### Nanoemulsion increased SOD activity in the rats’ synovial tissue

The MIA injection significantly decreased SOD activity in the rats’ synovial tissue (*P* < 0.001) (Fig. 2b). Although administration of the nanoemulsion gel, nanoemulsion solution, essential oils gel and diclofenac sodium gel significantly elevates SOD activity in the rats’ synovial tissue (*P* < 0.05), but the diclofenac sodium gel elevates SOD activity more than the other treatments.

#### Nanoemulsion increased GPx activity in the rats’ synovial tissue

As shown in Fig. 2c, the intra-articular injection of MIA caused a decrease in GPx activity in the rats’ synovial tissue compared to the CO group (*P* < 0.001). Although all treatments significantly increased the GPx activity compared to the OA group (*P* < 0.01), the application of the diclofenac sodium gel led to a prominent enhanced enzyme activity compared to other treatments.

### Histopathology

**Fig. 3:**
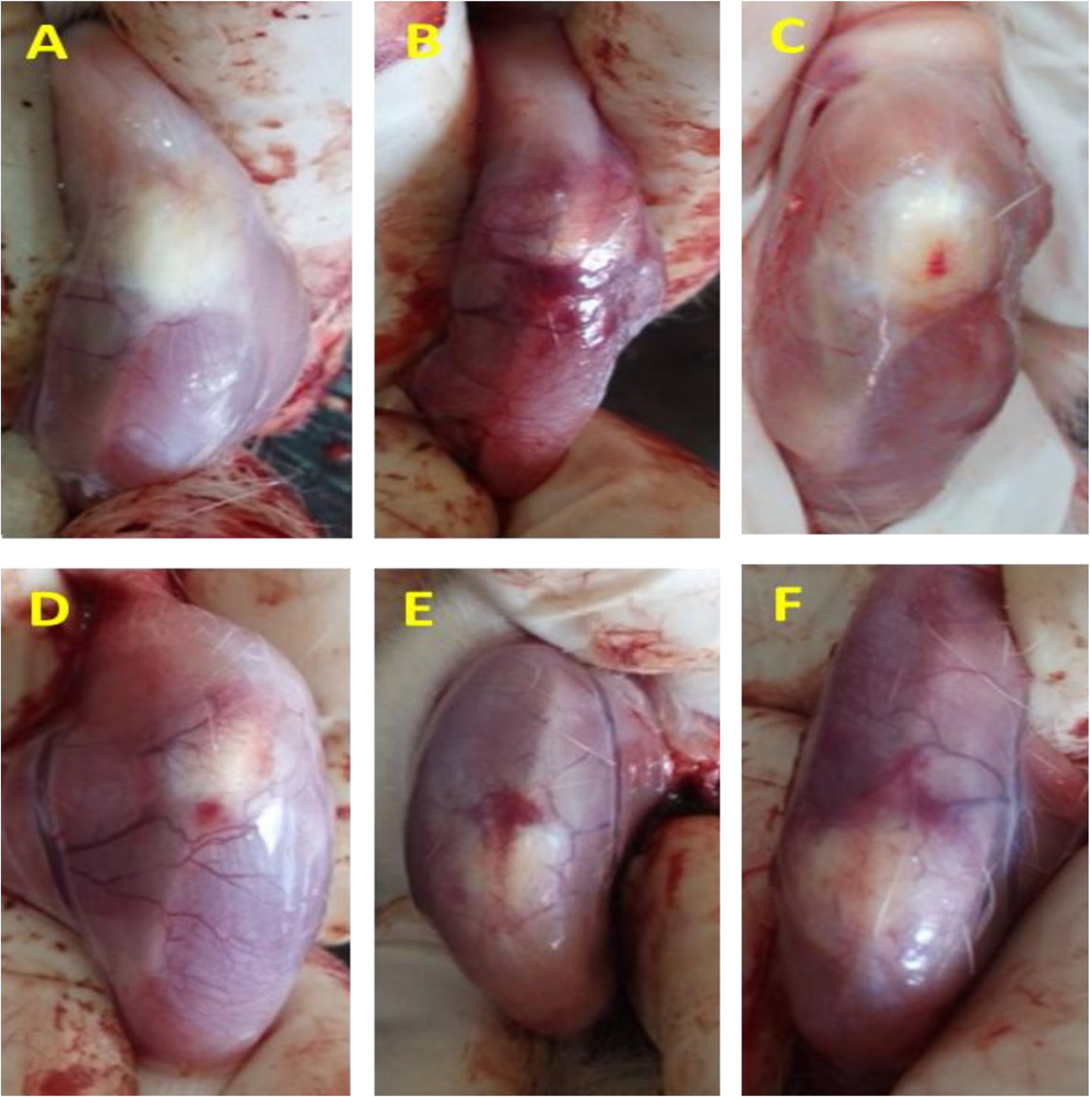
Macroscopic changes of the animal’s knee joint at the 14th day after intra-articular injection (*n* = 3 in each group). **a** The control group (no change), **b** the osteoarthritis group (severe hypertension and tissue analysis), **c** the osteoarthritis received gel contained the nanoemulsion group, **d** the osteoarthritis received gel contained Mentha and Rosemary essential oils group, **e** the osteoarthritis received the nanoemulsion solution group, and **f** the osteoarthritis received gel contained 1% diclofenac sodium group

**Fig. 4:**
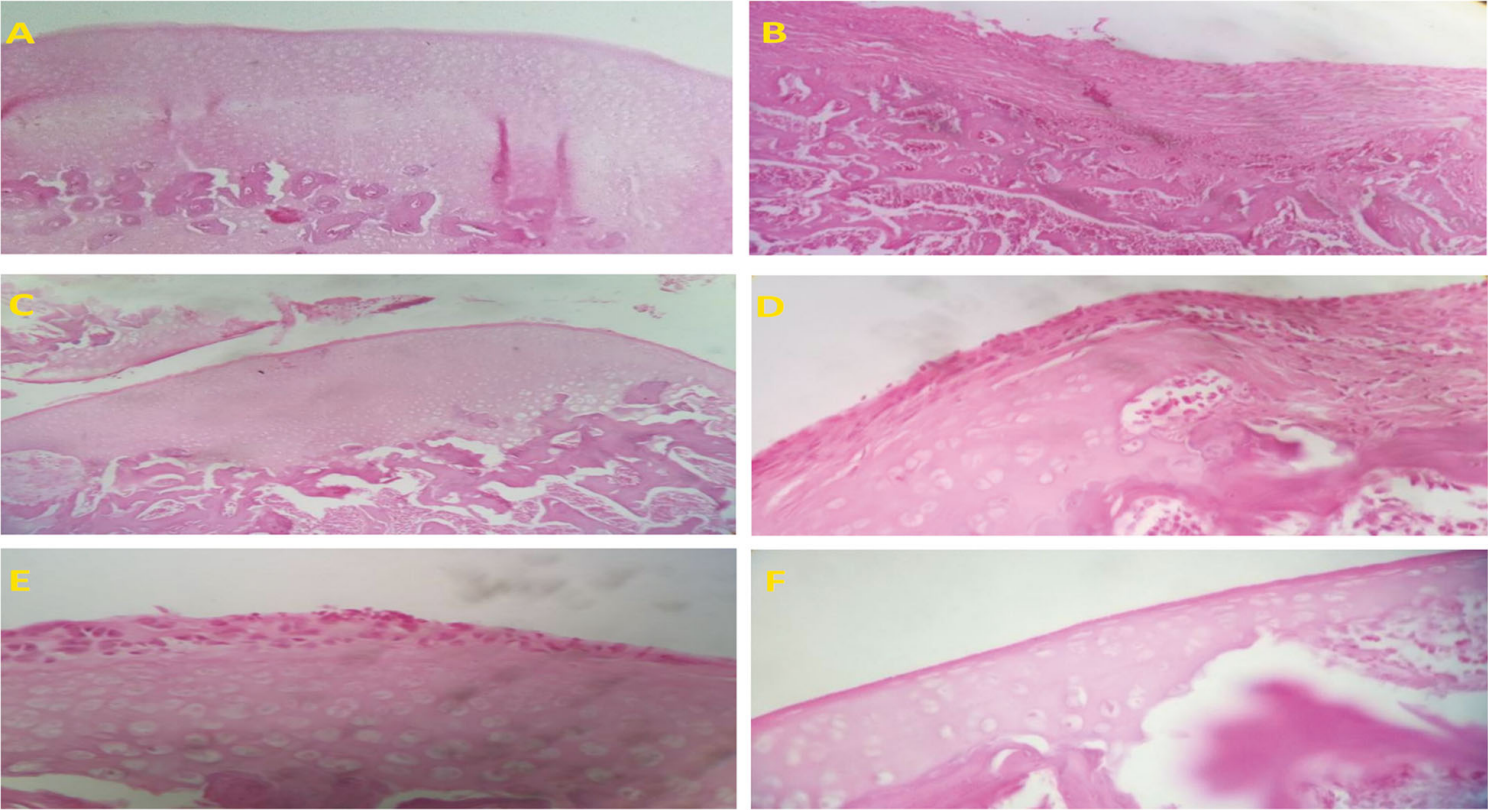
Microscopic images of articular tissue of rats at the 14th day after intra-articular injection (*n* = 3 in each group). Hematoxylin and Eosin staining, 100X. **a** The control group (no change), **b** the osteoarthritis group (severe hypertension and tissue analysis), **c** the osteoarthritis received gel contained the nanoemulsion group, **d** the osteoarthritis received gel contained Mentha and Rosemary essential oils group, **e** the osteoarthritis received the nanoemulsion solution group, and **f** the osteoarthritis received gel contained 1% diclofenac sodium group

The pathological findings are represented in Table 3. The control rats that received intra-articular saline showed no change in the knee joint after 14 days, i.e., the articular tissue structures, including the synovial tissue, synovium, adjacent ligaments, tendons, and tendon sheath, and subcutaneous tissues, had typical tissue architecture. Chronic inflammatory reactions accompanied by tendon adhesion were observed in the MIA injected animals. Also, the formation of granulation tissue with neovascularization, edema, infiltration of mononuclear was noticed. Moreover, chondrocyte necrosis with loss of matrix caused a decrease in the articular cartilage thickness and led to the separation of the necrotic cartilage from the subchondral bone. In the NG and DG animals, inflammatory cell infiltration and cartilage injury significantly declined. In both groups’ animals, a few scattered mononuclear cells were observed, and the cartilage thickness increased compared to the OA group (Figs. 3 and 4).

**Table 3:** Comparison of histological features of different study groups

Groups	Pathological finding
Control (CO)	No cartilage degeneration; intact surface
Osteoarthritis (OA)	Severe cartilage degeneration (greater than 75% affected); formation of granulation tissue with neo vascularization; edema; infiltration of mononuclear leucocytes
Osteoarthritis received gel contained the nanoemulsion (NG)	Mild cartilage degeneration (11–25%); surface fibrillation; a few scattered lymphocytes; a very mild edema
Osteoarthritis received the nanoemulsion solution (NS)	Moderate cartilage degeneration (26–50%); moderate fibrillation; infiltration of mononuclear leucocytes
Osteoarthritis received gel contained Mentha and Rosemary essential oils (EG)	Marked cartilage degeneration (51–75%); neo vascularization; edema; infiltration of mononuclear leucocytes
Osteoarthritis received gel contained 1% diclofenac sodium (DG)	Mild cartilage degeneration (11–25%); surface fibrillation; a few scattered lymphocytes

## Discussion

The present study aimed to investigate the effect of a nanoemulsion containing peppermint and rosemary essential oils in an animal model of osteoarthritis. Since we used this nanoemulsion as a topical medication to relieve induced joint pain, evaluation of dermal toxicity, and dermal irritation was the first step of this study. Our results showed that the topical application of this nanoemulsion in male and female rats was not toxic. Comparing the lipid profile in the CO and NG groups showed that the nanoemulsion did not alter the lipid profile. Also, serum levels of the NG group’s aminotransferase enzymes, as markers of liver function, did not significantly change compared to the controls. Besides, no change in serum creatinine and urea after receiving the nanoemulsion indicated the animal’s kidney’s normal activity. The examination of hematologic factors showed that the nanoemulsion did not cause blood cell death and did not alter erythrocyte parameters such as hemoglobin and hematocrit. Based on our knowledge, there is no study to evaluate the effect of acute dermal treatment of a nanoemulsion on the hematological and biochemical parameters in an animal model. However, the Ribeiro et al. study has shown that oral administration of nanoemulsion containing *Eucalyptus staigeriana* essential oil did not alter the rats’ hematological parameters after the treatment [25]. Ragavan and colleagues have shown that acute oral treatment of garlic oil nanoemulsion did not change renal markers such as urea and creatinine and hematologic factors of rats [26]. Also, we did not observe any dermal reactions after applying the nanoemulsion gel on the rabbit skin. Researchers have shown that nanoemulsion containing natural origin drugs had no dermal toxicity and can lead to better interaction with the skin and enhance skin penetration [27]. Mou et al. have demonstrated that nanoencapsulation of menthol and camphor provides better interaction with the skin and permeation enhancing ability and might be a promising carrier for topical drug delivery [28]. Furthermore, Sugumar and colleagues made a nanoemulsion containing eucalyptus oil, which had no irritancy and improved the wound healing process than the neomycin treated rats [29].

In the second part of the study, we induced osteoarthritis by intra-articular injection of MIA to evaluate the nanoemulsion’s analgesic effect. Previous studies showed that MIA injection into the knee joint causes chondrocyte apoptosis [30], which results in an imbalance in the extracellular matrix and cartilage damage leading to the OA progression [31]. The MIA injection to the joint leads to the release of peripheral pro-inflammatory mediators and neuropeptides such as prostaglandins, substance P, and bradykinin [30]. This inflammatory response can induce sensitization of the nociceptive receptor and hyperactivates the pain pathways [32]. Our results showed that all the treatments improve the cartilage change induced by MIA injection and reduce the joint pain. Also, the nanoemulsion and diclofenac, an analgesic and anti-inflammatory drug for relieving pain in many inflammatory diseases were more efficient.

Studies have previously shown that rosemary and peppermint essential oils have an analgesic effect. Ghannadi et al. demonstrated that combined rosemary and lavender essential oil ointment reduces pain and improves physical function in patients with knee osteoarthritis [33]. Another study showed that rosemary essential oil inhibits pain during both neurogenic and inflammatory phases of the formalin test, reduces the number of acetic acid-induced writhing, and inhibits the edema formation after carrageenan injection in the rat paw edema test [34]. Atta and Alkofahi have confirmed the anti-nociceptive and anti-inflammatory effects of peppermint. Their results demonstrated that peppermint could delay the reaction time to the hot-plate-induced thermal stimulation and inhibit acetic acid-induced writhing [35]. These findings were consistent with another study showing that the peppermint leaf extract has significant analgesic effects against thermal stimulation and writhing tests in mice [7]. The more reducing pain in the NS in comparison to the EG group can be attributed to the small size of nano-droplets. Also, polyethylene glycol in nanoemulsion structure can bind to the active ingredients and results in their controlled release. Moreover, the presence of tween 20 and tween 80 in its structure reduces the surface tension between the nanoemulsion’s components and water [36].

Previous studies have indicated the role of oxidative stress and inflammatory reactions in degenerative processes in OA. Jiang et al. demonstrated that chondrocytes apoptosis is mitochondrial-dependent and related to reactive oxygen species (ROS) elevation [37]. Ashkavand et al. reported that the pro-inflammatory mediators such as interleukin 1-B and nitric oxide elevates in animals received MIA [30]. Their results also showed that MDA content in the articular tissue increases, indicating that lipid peroxidation increases after pro-inflammatory mediators elevation [30]. The SOD activity is representing the ability of the body to scavenge free radicals. Gan et al. have demonstrated that the SOD activity positively correlates with the severity of damage in osteoarthritis disease [38]. Our results are inconsistent with previous studies. All applied intervention significantly decreased the MDA level and increased SOD and GPx activity compared to the OA group. Based on our knowledge, although no study evaluates the antioxidant effect of peppermint and rosemary essential oils in osteoarthritis, it has been previously proven in the other disorders. For example, Khalil et al. have shown that peppermint essential oil decreases the MDA and increases the activity of SOD and GSH in the liver exposed to CCL4 [39]. Also, Roskovic et al. have reported that rosemary essential oil can normalize the elevated MDA amount and reverse the oxidative stress-related enzymes in rats receiving CCL4 [12]. Furthermore, Hussein and colleagues have demonstrated that nanoencapsulation increased the rosemary essential oil’s stability and antioxidant activity [40].

In line with our results, Belkhodja et al. have shown that the rosemary essential oil reduces osteoarthritis histological score in rats with MIA injection [13]. Their results have demonstrated that although rosemary decreases the degradation of articular cartilage and inflammation in the joint, but causes surface fibrillation and deep fissures in cartilage. Moreover, rosemary essential oil reduces the number of migrated cells and the volume of inflammatory exudates after intrapleural injection of carrageenan in rats [41].

One of this study’s future aims was to evaluate the nanoemulsion’s efficacy in patients with osteoarthritis. We loaded the nanoemulsion in a gel formulation to easily use and control the drug’s applied amount. To investigate gel formulation’s effect on the nanoemulsion efficacy, we added the NS group to the study groups. Our results demonstrated the difference between NG and NS group was not significant in none of the tests. We can conclude that the formulation of the nanoemulsion in the gel doesn’t change the effect of nanoemulsion on behavioral, biochemical, and histopathologic parameters.

## Conclusions

Our findings showed that the nanoemulsion containing peppermint and rosemary essential oil did not cause any irritation in the rabbit skin and any dermal toxicity and change in biochemical and hematological parameters in male and female rats. Moreover, the nanoemulsion reduced mechanical and thermal allodynia, thermal hyperalgesia, and ambulatory-evoked pain via increasing SOD and GPx activity, decreasing MDA levels, and improving the pathological features of rats’ knee joint with osteoarthritis. In conclusion, this novel nanoemulsion formulation can escalate the therapeutic effect of rosemary and peppermint essential oil as a topical treatment for osteoarthritis.

## Data Availability

The datasets during and/or analyzed during the current study available from the corresponding author on reasonable request.

## Abbreviations

OA: Osteoarthritis
MIA: Monosodium iodoacetate
TBA: Thiobarbitoric acid
SOD: Superoxide dismutase
GPx: Glutathione peroxidase
EDTA: Ethylene-diaminetetrachloroacetic acid
ROS: Reactive oxygen species
PWT: Paw withdrawal threshold
PWF: Paw withdrawals frequencies
PWL: Paw withdrawal latencies

## References

[1] Goldring MB, Goldring SR (2010). Articular cartilage and subchondral bone in the pathogenesis of osteoarthritis. Ann N Y Acad Sci.

[2] Sofat N, Ejindu V, Kiely P (2011). What makes osteoarthritis painful? The evidence for local and central pain processing. Rheumatology (Oxford).

[3] Zhang RX, Ren K, Dubner R (2013). Osteoarthritis pain mechanisms: basic studies in animal models. Osteoarthr Cartil.

[4] Martel-Pelletier J, Barr AJ, Cicuttini FM, Conaghan PG, Cooper C, Goldring MB (2016). Osteoarthritis. Nat Rev Dis Primers.

[5] Gardiner P. Peppermint (Mentha piperita): Longwood Herbal Task Force; 2000. http://www.mcp.edu/herbal

[6] Yadegarinia D, Gachkar L, Rezaei MB, Taghizadeh M, Astaneh SA, Rasooli I (2006). Biochemical activities of Iranian Mentha piperita L. and Myrtus communis L. essential oils. Phytochemistry.

[7] Taher YA. Antinociceptive activity of Mentha piperita leaf aqueous extract in mice. Libyan J Med. 2012;7(1). 10.3402/ljm.v7i0.16205. 10.3402/ljm.v7i0.16205 PMC3316159 22468149

[8] Kehili S, Boukhatem MN, Belkadi A, Ferhat MA, Setzer WN (2020). Peppermint (Mentha piperita L.) essential oil as a potent anti-inflammatory, wound healing and anti-nociceptive drug. Eur J Biol Res.

[9] Belemkar S, Thakre SA, Pata MK (2013). Evaluation of anti-inflammatory and analgesic activities of methanolic extract of Adhatoda vasica Nees and Mentha piperita Linn. Inventi Rapid Ethnopharmacol.

[10] Wadnap N, Johnson J, Bhatt N, Chitre D (2006). Efficacy and safety of RA-11 (O)–a herbal analgesic cream. Indian J Tradit Knowl.

[11] González-Trujano M, Peña E, Martínez A, Moreno J, Guevara-Fefer P, Deciga-Campos M (2007). Evaluation of the antinociceptive effect of Rosmarinus officinalis L. using three different experimental models in rodents. J Ethnopharmacol.

[12] Raskovic A, Milanovic I, Pavlovic N, Milijasevic B, Ubavic M, Mikov M (2015). Analgesic effects of rosemary essential oil and its interactions with codeine and paracetamol in mice. Eur Rev Med Pharmacol Sci.

[13] Belkhodja H, Meddah B, Meddah TirTouil A, Slimani K, Tou A (2017). Radiographic and histopathologic analysis on osteoarthritis rat model treated with essential oils of Rosmarinus officinalis and Populus alba. Pharm Sci.

[14] Ghasemzadeh MR, Amin B, Mehri S, Mirnajafi-Zadeh SJ, Hosseinzadeh H (2016). Effect of alcoholic extract of aerial parts of *Rosmarinus officinalis* L. on pain, inflammation and apoptosis induced by chronic constriction injury (CCI) model of neuropathic pain in rats. J Ethnopharmacol.

[15] Scalfo F, Davis S, Lai A, Karsdal M, Offord E, Ameye LG (2009). Rosemary extract slows down cartilage degeneration in bovine articular cartilage explants. J Hum Nutr Diet.

[16] São Pedro A, Santo I, Silva C, Detoni C, Albuquerque E. The use of nanotechnology as an approach for essential oil-based formulations with antimicrobial activity. In: Méndez-Vilas A, editor. Microbial pathogens and strategies for combating them, vol. 2. Badajoz: Formatex Research Center; 2013. p. 1364–74.

[17] Bilia AR, Guccione C, Isacchi B, Righeschi C, Firenzuoli F, Bergonzi MC (2014). Essential oils loaded in nanosystems: a developing strategy for a successful therapeutic approach. Evid Based Complement Alternat Med.

[18] Shah P, Bhalodia D, Shelat P. Nanoemulsion: a pharmaceutical review. Syst Rev Pharm. 2010;1(1):24–32.

[19] OECD. Test no. 404: acute dermal irritation/corrosion: OECD Publishing; 2002.

[20] OECD (2015). Test no. 402: acute dermal toxicity, OECD guideline for testing of chemicals (draft updated Test Guideline 402 on Acute Dermal Toxicity).

[21] Vonsy JL, Ghandehari J, Dickenson AH (2009). Differential analgesic effects of morphine and gabapentin on behavioural measures of pain and disability in a model of osteoarthritis pain in rats. Eur J Pain.

[22] Hamidi GA, Ramezani MH, Arani MN, Talaei SA, Mesdaghinia A, Banafshe HR (2012). Ethosuximide reduces allodynia and hyperalgesia and potentiates morphine effects in the chronic constriction injury model of neuropathic pain. Eur J Pharmacol.

[23] Niehaus WG, Samuelsson B (1968). Formation of malonaldehyde from phospholipid arachidonate during microsomal lipid peroxidation. Eur J Biochem.

[24] Markwell MA, Haas SM, Bieber LL, Tolbert NE (1978). A modification of the Lowry procedure to simplify protein determination in membrane and lipoprotein samples. Anal Biochem.

[25] Ribeiro WL, Camurça-Vasconcelos AL, Macedo IT, dos Santos JM, de Araújo-Filho JV, Ribeiro Jde C (2015). In vitro effects of Eucalyptus staigeriana nanoemulsion on Haemonchus contortus and toxicity in rodents. Vet Parasitol.

[26] Ragavan G, Muralidaran Y, Sridharan B, Nachiappa Ganesh R, Viswanathan P (2017). Evaluation of garlic oil in nano-emulsified form: optimization and its efficacy in high-fat diet induced dyslipidemia in Wistar rats. Food Chem Toxicol.

[27] Rai VK, Mishra N, Yadav KS, Yadav NP (2018). Nanoemulsion as pharmaceutical carrier for dermal and transdermal drug delivery: formulation development, stability issues, basic considerations and applications. J Control Release.

[28] Mou D, Chen H, Du D, Mao C, Wan J, Xu H (2008). Hydrogel-thickened nanoemulsion system for topical delivery of lipophilic drugs. Int J Pharm.

[29] Sugumar S, Ghosh V, Nirmala MJ, Mukherjee A, Chandrasekaran N (2014). Ultrasonic emulsification of eucalyptus oil nanoemulsion: antibacterial activity against Staphylococcus aureus and wound healing activity in Wistar rats. Ultrason Sonochem.

[30] Ashkavand Z, Malekinejad H, Amniattalab A, Rezaei-Golmisheh A, Vishwanath B (2012). Silymarin potentiates the anti-inflammatory effects of Celecoxib on chemically induced osteoarthritis in rats. Phytomedicine.

[31] Chiu PR, Hu YC, Huang TC, Hsieh BS, Yeh JP, Cheng HL, et al. Vitamin C protects chondrocytes against monosodium iodoacetate-induced osteoarthritis by multiple pathways. Int J Mol Sci. 2016;18(1):38. 10.3390/ijms18010038 PMC5297673 28035982

[32] Fernihough J, Gentry C, Malcangio M, Fox A, Rediske J, Pellas T (2004). Pain related behaviour in two models of osteoarthritis in the rat knee. Pain.

[33] Ghannadi A, Karimzadeh H, Tavakoli N, Darafsh M, Ramezanloo P (2013). Efficacy of a combined rosemary and lavender topical ointment in the treatment of patients with osteoarthritis of the knee. Zahedan J Res Med Sci.

[34] Faria L, Lima CS, Perazzo FF, Carvalho JCT. Anti-inflammatory and antinociceptive activities of the essential oil from *Rosmarinus officinalis* L.(Lamiaceae). Int J Pharm Sci Rev Res. 2011;7(2):1–8.

[35] Atta A, Alkofahi A (1998). Anti-nociceptive and anti-inflammatory effects of some Jordanian medicinal plant extracts. J Ethnopharmacol.

[36] Alam P, Ansari MJ, Anwer MK, Raish M, Kamal YK, Shakeel F (2017). Wound healing effects of nanoemulsion containing clove essential oil. Artif Cells Nanomed Biotechnol.

[37] Jiang L, Li L, Geng C, Gong D, Jiang L, Ishikawa N (2013). Monosodium iodoacetate induces apoptosis via the mitochondrial pathway involving ROS production and caspase activation in rat chondrocytes in vitro. J Orthop Res.

[38] Gan HS, Tan TS, Wong LX, Tham WK, Sayuti KA, Abdul Karim AH (2014). Interactive knee cartilage extraction using efficient segmentation software: data from the osteoarthritis initiative. Biomed Mater Eng.

[39] Khalil AF, Elkatry HO, El Mehairy HF (2015). Protective effect of peppermint and parsley leaves oils against hepatotoxicity on experimental rats. Ann Agric Sci.

[40] Hussein AM, Kamil MM, Lotfy SN, Mahmoud KF, Mehaya FM, Mohammad AA (2017). Influence of nano-encapsulation on chemical composition, antioxidant activity and thermal stability of rosemary essential oil. Am J Food Technol.

[41] Takaki I, Bersani-Amado LE, Vendruscolo A, Sartoretto SM, Diniz SP, Bersani-Amado CA (2008). Anti-inflammatory and antinociceptive effects of Rosmarinus officinalis L. essential oil in experimental animal models. J Med Food.

